# Cytotoxic and Infection-Controlled Investigations of Novel Dihydropyridine Hybrids: An Efficient Synthesis and Molecular-Docking Studies

**DOI:** 10.3390/ph16081159

**Published:** 2023-08-15

**Authors:** Mallikarjuna R. Guda, Grigory. V. Zyryanov, Amit Dubey, Venkata Subbaiah Munagapati, Jet-Chau Wen

**Affiliations:** 1 Institute of Chemical Engineering, Ural Federal University Named after the First President of Russia B.N. Yeltsin, 28 Mira St., Yekaterinburg 620002, Russia; g.v.zyrianov@urfu.ru; 2 Department of Chemistry, Sri Venkateswara University, Tirupati 517502, India; 3 Ural Division of the Russian Academy of Sciences, I. Ya. Postovskiy Institute of Organic Synthesis, 22 S. Kovalevskoy Street, Yekaterinburg 620219, Russia; 4 Computational Chemistry and Drug Discovery Division, Quanta Calculus Pvt. Ltd., Greater Noida 201310, India; 5 Department of Pharmacology, Saveetha Dental College and Hospital, Saveetha Institute of Medical and Technical Sciences, Chennai 600077, India; 6 Research Centre for Soil and Water Resources and Natural Disaster Prevention (SWAN), National Yunlin University of Science and Technology, Douliou 64002, Taiwan; 7 Department of Safety, Health, and Environmental Engineering, National Yunlin University of Science and Technology, Douliou 64002, Taiwan

**Keywords:** 1,4-dihydropyridine, green synthesis, computer studies, MIC, MBC/MFC, cytotoxic assay

## Abstract

A sequence of novel 1,4-dihydropyridines (DHP) and their hybrids was developed using a multicomponent strategy under environmentally benign conditions. In addition, computational studies were performed, and the ligand–protein interactions calculated in different bacteria and two fungal strains. Para-hydroxy-linked DHP (**5f**) showed the best binding energies of 3.591, 3.916, 8.499 and 6.895 kcal/mol against various pathogens used and other substances received a good docking score. The pathogen resistance potential of the synthesized targets against four bacteria and two fungi showed that whole DHP substances exhibit different levels of resistance to each microorganism. Gram-positive bacteria, which are highly sensitive to all molecules, and the MTCC-1884-encoded fungus strongly rejected the studied compounds compared to comparator drugs. In particular, the **5f** candidate showed remarkable antimicrobial activity, followed by the substances **5a**, **5b**, **5j**, **5k** and **5l**. Furthermore, MIC and MBC/MFC properties showed that **5f** had a minimum bacterial concentration of 12.5 μg/mL against *E. coli* and against two fungal pathogens, with its killing activity being effective even at low concentrations. On the other hand, whole motifs were tested for their cytotoxic activity, revealing that the methoxy and hydroxy-linked compounds (**5h**) showed greater cytotoxic potency, followed by the two hydroxy linked compounds (**5d** and **5f**). Overall, this synthetic approach used represents a prototype for future nature-favored synthesis methods and these biological results serve as a guide for future therapeutic drug research. However, the computer results play an important role in the further development of biological experiments.

## 1. Introduction

The selection of a key chemical compound for synthesis is important because these substances must have shown therapeutic efficacy in previous studies. Numerous investigators have reported and proven that DHP-core-owning compounds exhibit diverse biological characteristics. Also, it is used in medicines such as amlodipine to treat blood pressure and angina (for chest pain), nifedipine–calcium channel blockers, as well as to lower blood pressure in the arteries when the heart is not working much, to maintain blood circulation, and in nicardipine has a similar effect in curing ailments and diseases [1,2,3]. This substance acts as an antituberculosis agent and plays a leading role in metabolic oxidation [4]. Recently, Gomha et. al. developed novel dihydropyridines that conferred antimicrobial properties. However, this study used a well-known conventional method. [5]. The regular production conditions for the production of 1,4-dihydropyridine analogues and these produced compounds showed antitumor properties [6]. Likewise, various types of research strategies have been applied for the production of pyridines via the acid-mediated condition [7], enzymatically involved synthesis [8,9] and the heavy metal initiator method [10], nano catalyst utilization reactions [11] and molybdenum-type metal oxide with catalysts [12]. All the above solutions have numerous disadvantages such as metals, toxic reagents, alloy oxides and others that are undesirable from a green chemistry perspective. 

Environmental sustainability remains a challenging aspect of all scientific investigations, particularly synthetic research [13,14,15]. Sometimes the molecule-production strategy does not fall under the twelve principles of green chemistry, resulting in [16] damage to nature from production waste. The eco-friendly synthetic approaches that support the manufacture of compounds should be designed to avoid waste and toxic pollution that affect nature and humans [17,18,19,20]. Leading portfolios of green manufacturing processes include aqua engagement synthesis, water-soluble solvent conditions, neat reactions, sonochemical techniques, multiple reactants involved in single-step reactions, microwave-heating pathways, and ball-milling strategies.

Besides synthesis and biological experiments, computational studies are becoming the crucial tool in organic research focusing on structure orientation, compound behavior, molecular properties prediction, ligand–protein interactions, binding energies, and others [21]. In recent years, numerous results related to machine-assisted studies combined with synthetic research have been published [22,23,24,25,26]. As far as we are aware, there are currently no reports combining information about DHP, its environmentally friendly manufacturing method, biological properties, and computer simulations. The present research involved the green synthesis approach, synthesized the bulk of the dihydropyridine substances and investigated their antimicrobial and cytotoxic potential. Computer-assisted characteristic curves are also discussed in detail.

## 2. Results and Discussion

### 2.1. Chemistry

**Scheme 1:**
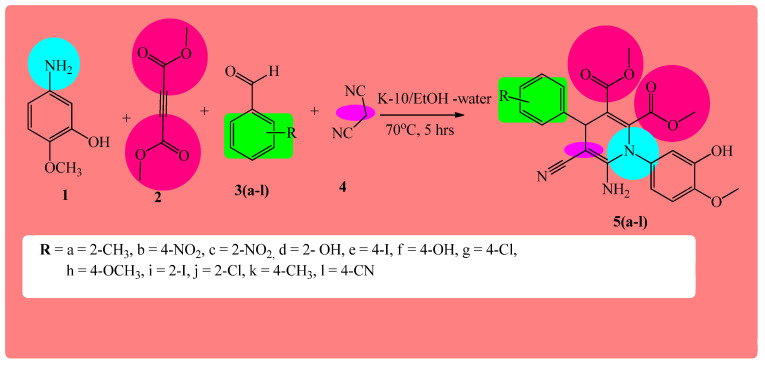
Single-step synthesis of dihydropyridine analogues.

As nature favors synthetic research, researchers are adopting strategies such as time saving, reactant minimization, simple solvents, one-step synthesis, cost savings, and volume yield to protect the environment. In this context, to identify the best portfolio in the area of green synthesis for the development of DHP compounds, a number of methods were used and are shown in Scheme 1 and Table 1. All processes were performed in a one-pot, multicomponent reaction using 5-amino-2-methoxyphenol, dimethylbut-2-ynedioate, several aldehydes, and malononitrile as starting reactants. It is believed that the commonly available polyethylene glycol (PEG) contemplates the production of compounds **5a**, **5b** and **5c** to maintain economically viable conditions. The synthesis began when the starting chemicals of product **5a** were added to PEG and gently heated to elevated temperatures. Product **5a** was obtained in about 45 percent, followed by products **5b** and **5c** in yields of 39 and 41 percent, respectively, when their respective starting materials were involved in a reaction. After unexpected yields, various acid catalysts have been employed to achieve a profitable amount of end goals. In this manner, Lewis acid zinc chloride acid initiator was used under acetonitrile solvent conditions to prepare compounds **5a–c**. As a result, an unsatisfactory product yield was observed (Entry 2, Table 1).

In the same solvent medium, we changed the catalyst and continued the reactions, resulting in a trace yield of compounds with values of 11% (**5a**), 19% (**5b**), and 15% (**5c**) in the case of indium chloride as a catalyst (Entry 3, Table 1), while the product **5a** has a 32 percent yield in tin chloride promoter media, **5b** has a 25% yield, and the percentage of **5c** is about 21 (Entry 4, Table 1). Apart from a variety of acidic montmorillonite K-10 acid clay was considered a promoters for the production of **5a–c** units using a mixed-solvent concept. The clay initiator, the 2:1 miscible ethanol–water solvent combination and all of the reactants of **5a** were heated in an autoclave under argon conditions to yield the target **5a** in an amount of 86%. When the reactions of **5b** and **5c** were successfully carried out using the same strategy, the yields of these targets were 83 percent and 88 percent, showing a high productivity (Entry 5, Table 1). Thus, with this environmentally benign reaction strategy, the products (5(d–l)) were prepared in impressive yields.

### 2.2. Molecular Modelling

#### 2.2.1. Molecular-Docking Studies, Binding Pose, and Interaction Profiling

##### Molecular-Docking Studies

The interaction at the molecular level between the ligands and protein receptors was determined using molecular docking. The selected ligands were docked to the binding region of the germs *Bacillus subtilis*, *Staphylococcus aureus*, ATCC 8739 code bacteria, Proteus vulgaris, MTCC 1881 and Aspergillus flavus using Glide (Standard Precision Mode) in the Glide module [27]. The exact coordinates of the active sites of all protein receptors were determined using the Schrödinger grid generation method. To allow more accurate predictions for the ligand positions, using several sets of specifications, the receptors characteristics and shape were taken into consideration in a grid. The essential zone of the lattice, which designates the residue as the lattice center, should be covered by it [28,29,30,31,32]. Additional default settings were used. The optimal docking conformation of each ligand with the lowest docking score was considered.

##### Binding Poses and Interaction Profiling

The best docking scores for bond conformations were determined after molecular docking and compared. Furthermore, the default settings of the academic A Schrödinger-Maestro v11.4 suite were used to study molecular interactions at a distance of 4 Ao around the docked ligand (Release 2018-3 from Schrödinger: Maestro, Schrödinger, LLC, 2018, New York, NY, USA). For docked ligands and receptors, intermolecular interactions like cation, hydrophobic and hydrogen-bonding interactions that are not covalent have been identified in discovered negative contacts, positive contacts, glycine interactions and the formation of salt bridges.

#### 2.2.2. *Bacillus subtilis*

##### Binding Mode of Best Hit Ligands **5a** and **5f** with *Bacillus subtilis*

**Figure 1:**
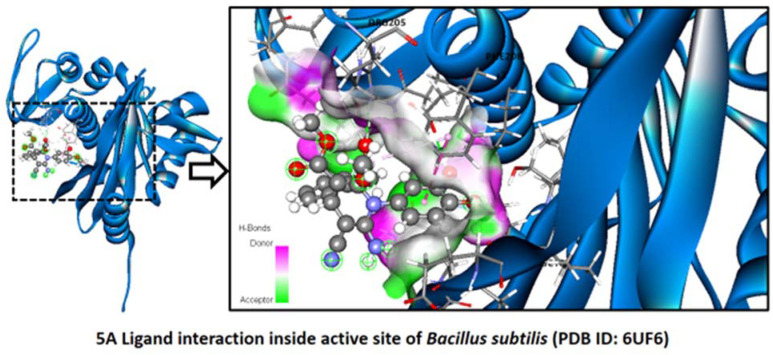
The molecular interactions and binding modes of best-hit ligand **5a** (ball–stick structure) at the binding site of *Bacillus subtilis* protein (blue cartoon structure).

**Figure 2:**
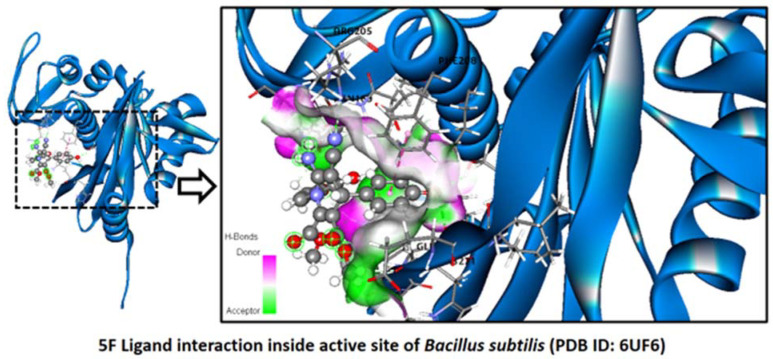
The molecular interactions and binding modes of best-hit ligand **5f** (ball–stick structure) at the binding site of *Bacillus subtilis* protein (blue cartoon structure).

The best projected binding energy for ligand **5a** is −3.593 kcal/mol. In the active site of *Bacillus subtilis*, this molecule and essential amino acids have created numerous, robust connections (Figure 1). For example, the amino acids Arg205 and Asp212 can interact three times with the ligand **5a** (Table 2). This ligand promotes additional receptor contacts (hydrophobic interactions, polar interactions) by building new connections. With −3.591 kcal/mol, ligand **5f** has the second-best binding energy. Ligand **5f** was reported to have developed some strong interactions with key amino acids in the Bacillus active site (Figure 2). For example, ligand **5f** may interact three times with Ser103, Arg205, Gln270, and Hip271 (Table 2). New interactions can arise as a result of increased contacts between the ligand and receptor (hydrophobic interactions, polar interactions).

#### 2.2.3. *Staphylococcus aureus*

##### Binding Mode of Best Ligands **5f** and **5j** with *Staphylococcus aureus*

**Figure 3:**
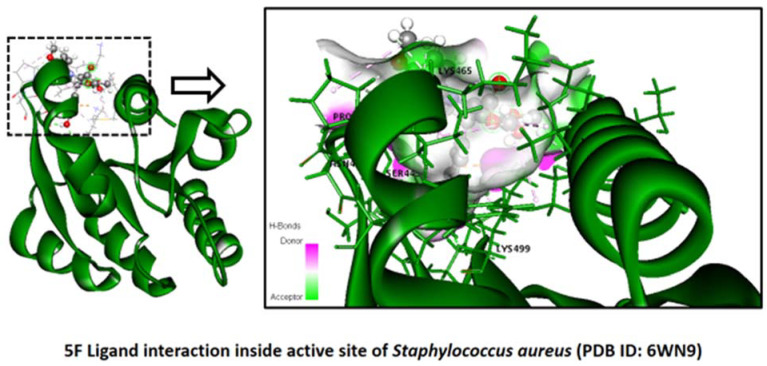
The molecular interactions and binding modes of best-hit ligand **5f** (ball–stick structure) at the binding site of *Staphylococcus aureus* protein (green cartoon structure).

**Figure 4:**
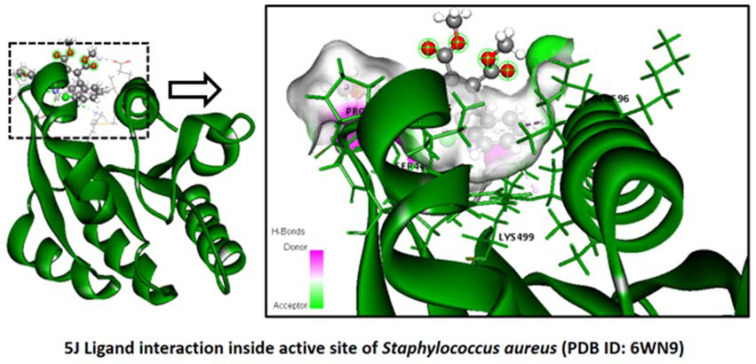
The molecular interactions and binding modes of best-hit ligand **5j** (ball–stick structure) at the binding site of protein (green cartoon structure).

One of the best ligands, **5f**, has a binding energy of −3.916 kcal/mol. The *Staphylococcus aureus* active site has evolved numerous strong connections between this molecule and important amino acids (Figure 3). For example, ligand **5f** may interact three times with amino acids Ser445, Asn468, and Lys499 (Table 3). This ligand creates novel connections that allow stronger hydrophobic and polar interactions with the receptor. Ligand **5j** has a binding energy of −3.919 kcal/mol. This ligand has formed several, strong interactions with key amino acids at the active site of *Staphylococcus aureus* (Figure 4). For example, ligand **5j** shows a threefold interaction with the amino acid Lys499 (Table 3). By forming new connections, this ligand allows more contacts (hydrophobic interactions, polar interactions) between the receptor *Staphylococcus aureus*.

#### 2.2.4. *Escherichia coli*

##### Binding Mode of Best Ligand **5k** and **5l** with *Escherichia coli*

**Figure 5:**
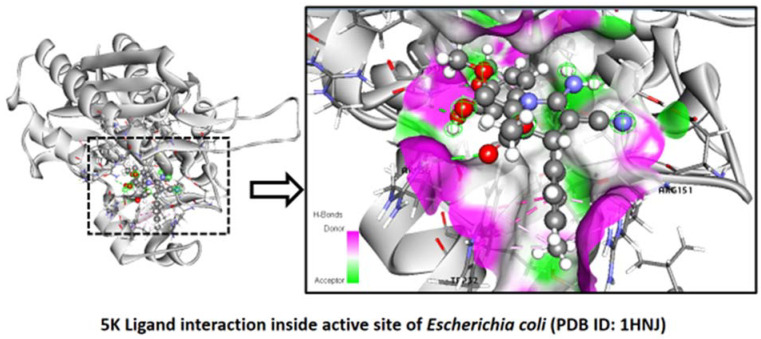
The molecular interactions and binding modes of best-hit ligand **5k** (ball–stick structure) at the binding site of *Escherichia coli* protein (grey cartoon structure).

**Figure 6:**
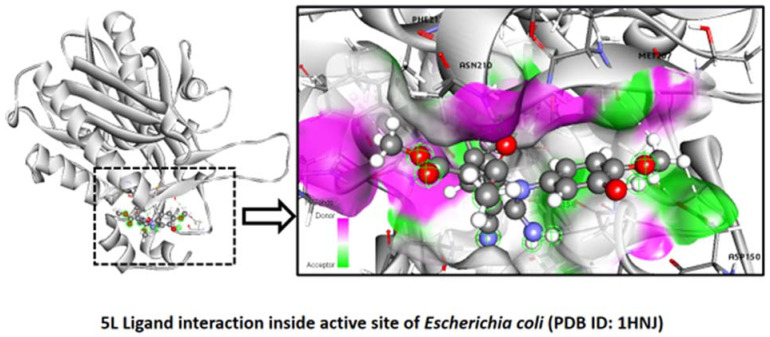
The molecular interactions and binding modes of hit ligand **5l** (ball–stick structure) at the binding site of *Escherichia coli* protein (grey cartoon structure).

With a calculated binding energy of −5.414 kcal/mol, ligand **5k** gave the best results by fully occupying the binding site. Some cluster residues, such as Asn247, show strong hydrogen bonding (Figure 5). The protein–ligand has also become more stable due to additional hydrophobic and polar interactions, as can be seen in Table 4. By fully occupying the binding site, **5l** achieved the best result with an estimated binding energy of −5900 kcal/mol. Some residues in clusters, such as Asn210, feature two hydrogen bonds (Figure 6). Furthermore, as shown in Table 4, additional hydrophobic interactions and polar interactions increased the stability of the protein–ligand complex.

#### 2.2.5. *Proteus vulgaris*

##### Binding Mode of Best Ligand **5f** and **5k** with *Proteus vulgaris*

**Figure 7:**
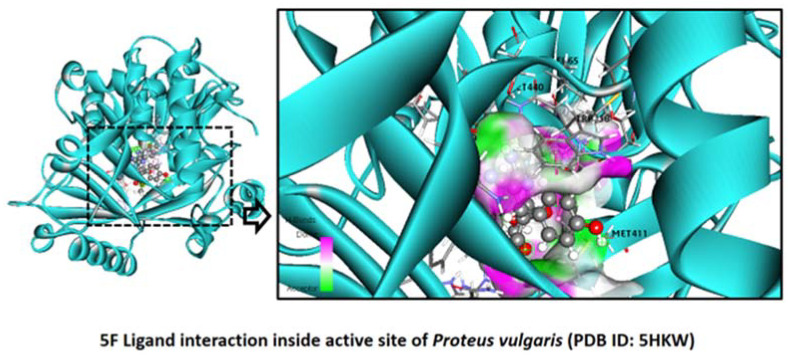
The molecular interactions and binding modes of hit ligand **5f** (ball–stick structure) at the binding site of *Proteus vulgaris* protein (cyan cartoon structure).

**Figure 8:**
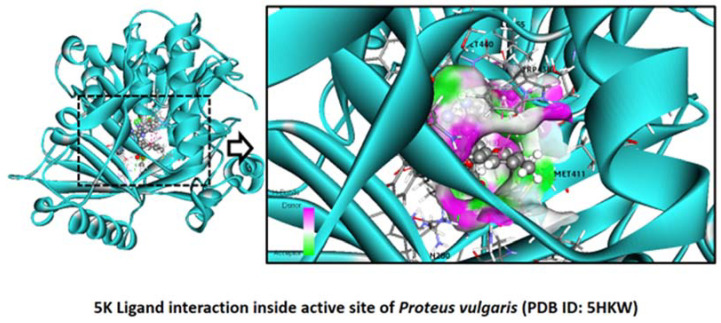
The molecular interactions and binding modes of hit ligand **5k** (ball–stick structure) at the binding site of *Proteus vulgaris* protein (cyan cartoon structure).

The best ligand **5f** has a binding energy of −8.499 kcal/mol. This ligand has formed several tight interactions with critical amino acids at the active site of *Proteus vulgaris* (Figure 7). For example, three hydrogen bonds were generated with amino acids Gln92, Gly98, and Gln99 by ligand **5f** (Table 5). Additional interactions (hydrophobic contacts, polar interactions, etc.) between the receptors are made possible by the shapes of these ligands. The second-best ligand **5k** has a binding energy of −8.322 kcal/mol. This molecule has formed several tight interactions with critical amino acids at the *Proteus vulgaris* active site (Figure 8). For instance, ligand **5k** can interact three times with amino acids Gln92, Tyr97, Gly98, and Gln99 through strong hydrogen bonding (Table 5). This ligand creates new interactions that allow the *Proteus vulgaris* receptors to make more contacts (hydrophobic interactions and polar interactions).

#### 2.2.6. *Aspergillus niger*

##### Binding Mode of Best Ligand **5b** and **5f** with *Aspergillus niger*

**Figure 9:**
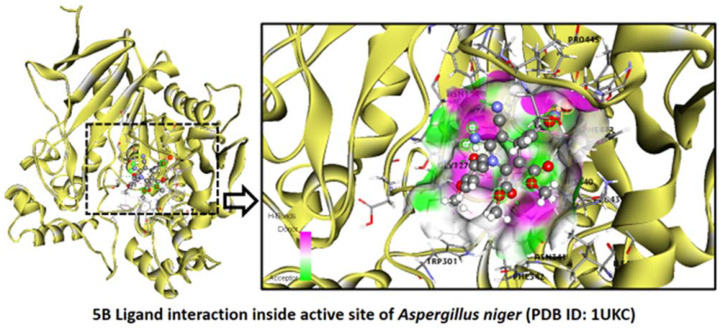
The molecular interactions and binding modes of hit ligand **5b** (ball–stick structure) at the binding site of *Aspergillus niger* protein (yellow cartoon structure).

**Figure 10:**
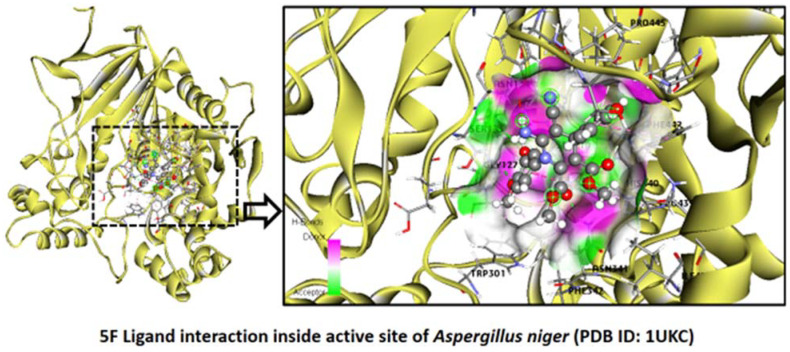
The molecular interactions and binding modes of hit ligand **5f** (ball–stick structure) at the binding site of *Aspergillus niger* protein (yellow cartoon structure).

With a binding energy of −6.828 kcal/mol, **5b** is the best ligand. At the active site of *Aspergillus niger*, this ligand has developed several significant interactions with important amino acids (Figure 9). Indeed, ligand **5b** can interact with amino acids, such as Asn134 and Asn341 through hydrogen bonding (Table 6). This ligand creates new connections that allow strong interactions with the receptors. The binding energy of the second-best ligand **5f** is −6.895 kcal/mol. At the *Aspergillus niger* active site, this molecule has developed numerous close contacts with crucial amino acids (Figure 10). Also, ligand **5f** can participate in hydrogen bonding with amino acids such as Asn134 and Asn341 (Table 6).

#### 2.2.7. *Aspergillus flavus*

##### Binding Mode of Best Ligand **5f** and **5h** with *Aspergillus flavus*

**Figure 11:**
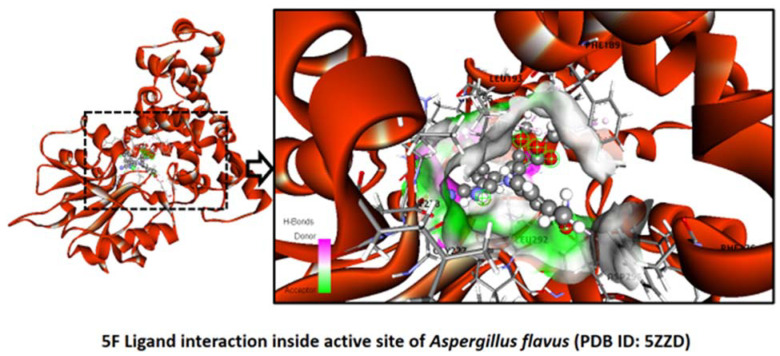
The molecular interactions and binding modes of best-hit ligand **5f** (ball–stick structure) at the binding site of *Aspergillus flavus* protein (red cartoon structure).

**Figure 12:**
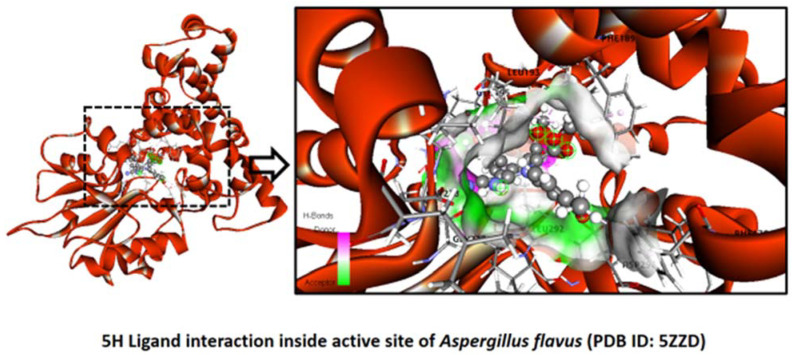
The molecular interactions and binding modes and of best-hit ligand **5h** (ball–stick structure) at the binding site of *Aspergillus flavus* protein (red cartoon structure).

The binding energy of the best ligand **5f** is −5.991 kcal/mol. At the *Aspergillus flavus* active site, this molecule, which contains an important amino acid, has created some powerful connections (Figure 11). For example, ligand **5f** can form three hydrogen bonds with amino acids Arg197, Gly227, and Asp296 (Table 7). This ligand allows additional contacts (polar and hydrophobic) between the receptors. The ideal ligand is **5h**, with a binding energy of −5.980 kcal/mol. In the active site of Aspergillus flavus, there are several strong interactions between ligand and critical amino acids. (Figure 12). For example, the amino acids Gly227 and Asp296 could form three separate hydrogen bonds with the ligand **5h** (Table 7). The unique connections this ligand makes allow for stronger hydrophobic and polar interactions with the receptor.

#### 2.2.8. Overall Docking Results and Discussion

**Figure 13:**
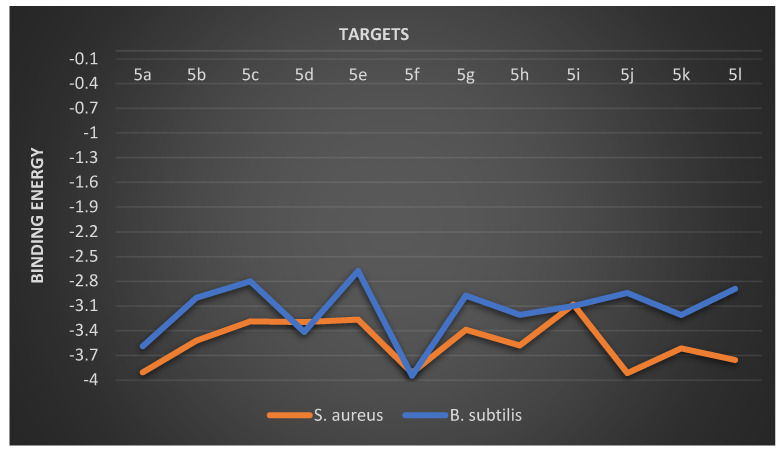
Binding energy score of prepared compounds against Gram-positive bacteria.

**Figure 14:**
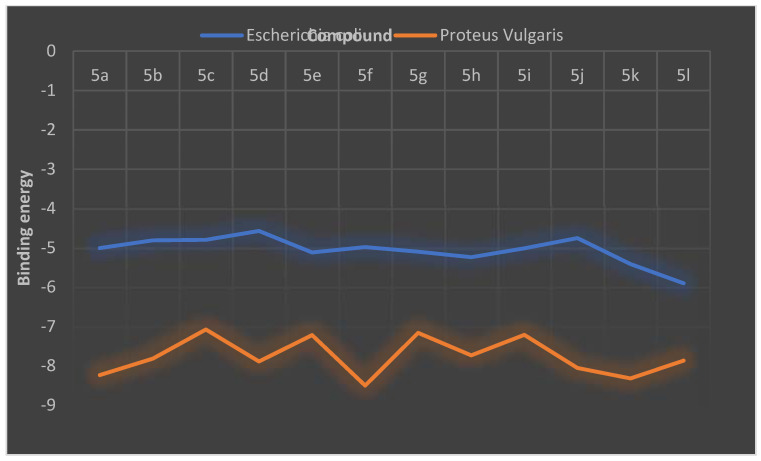
Binding energy score of prepared compounds against Gram-negative bacteria.

**Figure 15:**
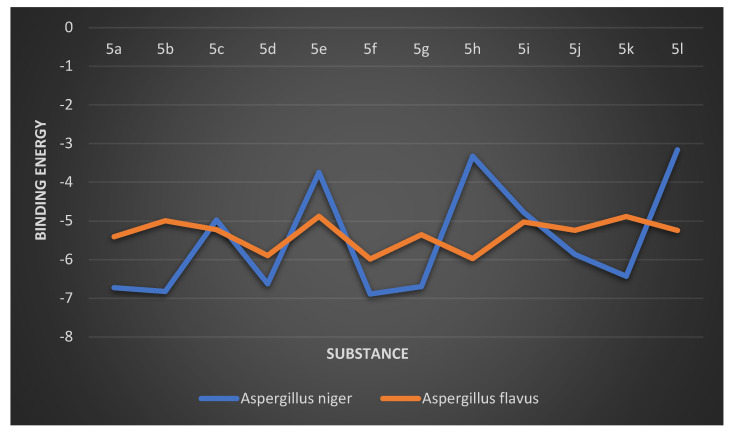
Binding energy score of prepared compounds against fungal germs.

The computational molecular-docking analysis of the targets revealed that the entire substances showed numerous interactions in binding energy regions of all pathogens (Figure 13, Figure 14 and Figure 15). Overall, **5f** targets show strong protein–ligand interactions in all docking studies except the machine-related *Escherichia coli* studies. The binding potential of this substance was 3.591 kcal/mol for *Bacillus subtilis*, and against *Staphylococcus aureus*, the value was 3.916 kcal/mol, 8.499 kcal/mol for *Proteus vulgaris* and 6.895 kcal/mol in the case against *Aspergillus niger* (Figure 13 and 14). Fascinatingly, in computer experiments with the pathogen *Escherichia coli*, the docking score had the highest value of all at 5.900 kcal/mol, which corresponds to **5l** (Figure 15). Furthermore, synthesized compound **5k** showed a possible protein–ligand interaction with a binding energy level of 5.414 kcal/mol, and since it has the second-best dock value, it is of importance. In fact, **5f** noted the most favorable binding energy value. Additionally, the remaining compounds showed a range of protein–ligand docking scores in the bacterial and fungal strains tested.

### 2.3. Biological Results 

#### 2.3.1. Antimicrobial Discussion

**Figure 16:**
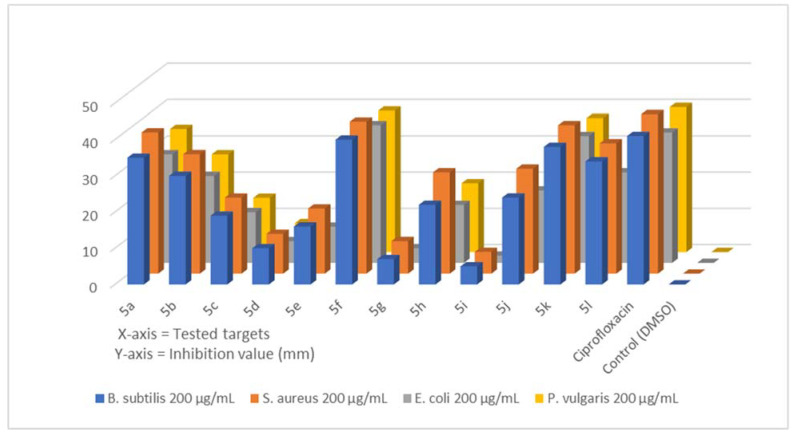
Targets’ resistance potential towards screened bacteria.

**Figure 17:**
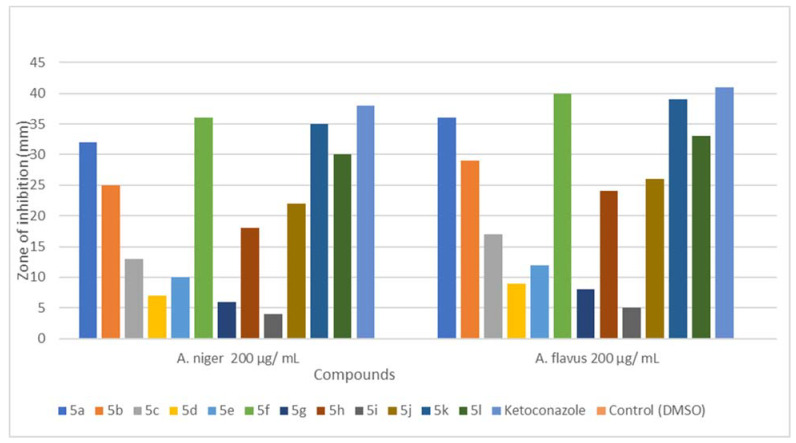
Targets’ resistance potential towards screened fungal.

The compounds and their antimicrobial effectiveness against various bacterial and fungal strains were measured using established methods. Ciprofloxacin is a drug used as a standard for bacterial resistance properties and the antifungal potential of the target molecules was compared to Ketoconazole drugs. To evaluate the biological activity of compounds, the bacteria ATCC 6633 (*Bacillus subtilis*), ATCC 8739 (*Escherichia coli*), ATCC 19,433 (*Staphylococcus aureus*), ATCC 29,213 (*Proteus vulgaris*) and the fungal MTCC 1881 (*Aspergillus niger*), MTCC-1884 (*Aspergillus flavus*) were used in this study. The results of the tested substances are listed in Table 8. From the data, dihydropyridine **5f** exposed higher levels of resistance to all four bacterial and two fungal strains than other compounds compared to the reference medicine. Its potential value is 40.12 mm in diameter at 200 μg/mL against *B. subtilis.*, for the ATCC 19,433 pathogen, a resistance area of 42.14 mm, 36.19 mm against *A. niger* and an inhibition zone of 40.14 mm in the case of the *A. flavus* fungus. Moreover, compounds **5a**, **5b**, **5j**, **5k** and **5l** then showed good activity against all pathogens. Although the whole tested motifs showed different resistance energies to all microbes used, the bacterial strains ATCC 6633 and ATCC 19,433 are more strongly attacked by entire tested substances than the other two bacteria. Also, in between fungal pathogens, the MTCC-1884 code strain shows stronger resistance to compounds than the *A. flavus* strain, which means that the screened compounds showed highly antifungal characteristics against *A. flavus.* On the other hand, **5c**, **5e**, and **5h** showed moderate potency in resistance to all pathogens, while the other compounds were showed mild potency in killing fungal and bacterial pathogens (Figure 16 and Figure 17).

#### 2.3.2. MIC and MBC/MFC Potency of Tested Compounds

Table 9 shows the test results of the smallest inhibitory dose (MIC) and smallest bacteria/fungal dose (MBC/MFC) of the five best compounds, which already showed a more pronounced zone of inhibition than other compounds against all pathogens. Among these, compound **5a** has since shown the lowest bacterial concentration (MBC) of 50 μg/mL, which is twice the MI concentration in *B. subtilies.* In fact, composite **5b** gave the lowest bacterial volume (MBC) (100 μg/mL) versus *E. coli* bacteria. This value corresponds to twice the MIC (50 μg/mL). Substances **5k** and **5l** had the lowest bacterial concentration characteristics against *E. coli* (25 and 50 μg/mL), which is twice the MI concentration. Further, it was found that the MB concentrations of **5f** and **5l** were twice the MI concentration using the *S. aureus* pathogen. Moreover, **5f** had the lowest fungal volume (MFC) of 50 and 100 μg/mL against all fungal pathogens, which was twice the MI concentration. The 100 μg/mL value of **5a** and the 25 μg/mL value of **5k** were found using the fungus *A. flavus.* Both of these results are double their MIC. In addition, the MF concentration in **5b** in the case of the pathogen *A. niger* is 2XMIC.

### 2.4. The In Vitro Cytotoxic Assay

The cytotoxic potency of the substances **5(a-l)** was tested using the MTT assay against the cell lines HepG2, U937 and SKOV3. Etoposide was used as a reference and Table 10 discloses the cytotoxicity results showing that the compounds showed promising cytotoxic activity against the HepG2 cancer cell line than the other two cell lines tested. Among all the test results of substances against the three cell lines, **5h** exhibited the most significant efficacy value of 29 μg/mL (HepG2), 36 (U937) and 51 μg/mL (SKOV3) compared to the standard cell line. Also, amalgams **5d**, **5f** and **5k** showed good effectiveness at IC50s; 57, 74, 88 μg/mL, 42, 60, 75 μg/mL and 59, 83, 95 μg/mL against three cell lines. While the ortho-methyl-substituted dihydropyridine (**5a**), **5g** (para-chloro attached DHP) and para-cyano linked DHP compound (**5l**) gave moderate biological potency compared to other motifs. In fact, in the case of the U937 and SKOV3 cell lines, the substance **5c** displayed a very weak therapeutic activity with more than 200 μg/mL, whereas the compounds **5e** and **5i** gave similarly lower activity towards SKOV3 at more than 200 μg/mL. Fortunately, motif **5c** resulted in an identifiable biological potency against HepG2 than the other two cell lines. Moreover, the other evaluated motifs showed some cytotoxicity related to the referee motif. The values of 86, 123, 160 μg/mL belong to **5b** and for **5j** to the IC_50_ values 84, 117, 129, respectively. 

**Figure 18:**
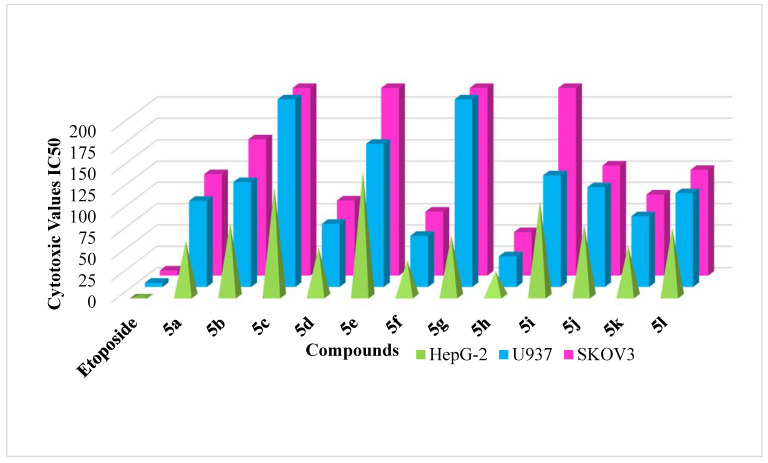
Targets’ cytotoxic potential towards screened cell line.

The structure–activity relationships are crucial for the relationship between the biologically active results and the targets tested. In this contradiction, most of the potency values of **5h** (Table 10) associated with other motifs could be due to the presence of a methoxy group as a substituted compound at the fourth position of benzene (Figure 18). Also, the amalgams **5d** and **5f** displayed the most cytotoxic results next to compound **5h**. The existence of a hydroxy group on the ring of benzene may be the cause of this. Moreover, the para-hydroxy benzene ring bearing the compound **5f** shows the best potency compared to motif **5d** bound to the ortho-hydroxybenzene ring. From the above statements, it can be concluded that the compounds linked with methoxy and hydroxy groups (**5h**) showed a greater therapeutic efficacy than the compounds linked with two hydroxy groups (**5d** and **5f**).

## 3. Materials and Methods

### 3.1. Way of Preparation for the Target Compounds **5(a–l)**

Malononitrile (**4**, 1 mmol) and methylbenzaldehyde (**3a**, 1 mmol) were added to an autoclave glassware that already had 7 mL of an ethanol–water solvent, and the mixture was stirred under environmental conditions for 30 min. After adding the aminomethoxyphenol (**1** 1mmol), dimethyl acetylenedicarboxylate (**2**, 1 mmol), and catalyst K-10, the process was conducted for 5 h at 70 °C. The target was obtained by means of crude filtration and extraction of crude with dichloromethane solvent followed by concentration. Utilizing 2-propanol, the color solid 5a was recrystallized. This technique achieved **5(b-l)** targets.

#### 3.1.1. Dimethyl-6-amino-5-cyano-4-(*o*-Tolyl)-1-(3-hydroxy-4-methoxyphenyl)-1,4-dihydropyridine-2,3-dicarboxylate (**5a**)

Amorphous yellowish green. M.p. 169–171; Yield 86 percent; NMR of proton in DMSO-*D*_6_ (*δ*) (300 MHz): = 7.15–7.17 (Doublet, 1H, aromatic), 7.23–7.39 (m, 4H, aromatic), 7.50–7.52 (Doublet, 1H, aromatic), 7.67 (Singlet, 1H, N-attached aryl hydrogen), 4.61 (s, 1H, dihydropyridine), 4.27 (s, 2H, NH_2_), 2.34 (s, 3H, Methyl), 3.91 (s, OCH_3_, 3H), 3.70 (s, OCH_3_, 3H), 3.43 (s, OCH_3_, 3H) ppm; NMR of carbon in CDCl_3_ + DMSO-*D_6_* (75 MHz): δ = 167.2 (Carbonyl) 164.1 (Carbonyl), 160.8 (Amine ‘C’), 149.5 (Ring N attached ‘C’), 143.4, 141.7, 140.5, 137.2, 135.0, 131.5, 128.4, 127.1, 125.3, 124.8, 121.1, 120.2, 117.8, 114.9 (CN), 62.5 (Methoxy), 60.9 (Methoxy), 57.5 (CN attached ‘C’), 55.4 (Methoxy), 36.7 (CH), 22.3 (Methyl) ppm. Molecular formula = C_24_H_24_N_3_O_6_[(M + H)^+^]: HRMS *m*/*z* = Analyzed (450.1665)/Identified (450.1662).

#### 3.1.2. Dimethyl-6-amino-5-cyano-4-(*p*-nitrophenyl)-1-(3-hydroxy-4-methoxyphenyl)-1,4-dihydropyridine-2,3-dicarboxylate (**5b**)

Amorphous yellow color. M.p. 202–204; Yield 83 percent; NMR of proton (300 MHz, DMSO-*D*_6_): δ = 7.04–7.07 (Doublet, aromatic, 1H), 7.26–7.33 (multiple, aromatic, 4H,), 7.47–7.49 (Doublet, 1H, aromatic), 7.64 (Singlet, 1H, N-attached aryl hydrogen), 4.66 (s, 1H, dihydropyridine), 4.11 (s, 2H, NH_2_), 3.81 (s, OCH_3_, 3H), 3.62 (s, OCH_3_, 3H), 3.50 (s, OCH_3_, 3H) ppm; NMR of carbon in CDCl_3_ + DMSO-*D*_6_ (75 MHz): δ = 167.6 (Carbonyl) 162.5 (Carbonyl), 160.2 (Amine ‘C’), 151.3 (Ring N attached ‘C’), 145.1, 143.5, 141.3, 138.6, 135.7, 133.0, 131.2, 128.5, 126.4, 123.1, 118.0, 115.2 (CN), 64.1 (Methoxy), 61.0 (Methoxy), 58.2 (CN attached ‘C’), 56.4 (Methoxy), 36.8 (CH) ppm. Molecular formula = C_23_H_21_N_4_O_8_[(M + H)^+^]: HRMS *m*/*z* = Analyzed (481.1359)/Identified (481.1361).

#### 3.1.3. Dimethyl-6-amino-5-cyano-4-(*o*-nitrophenyl)-1-(3-hydroxy-4-methoxyphenyl)-1,4-dihydropyridine-2,3-dicarboxylate (**5c**)

Amorphous pale yellow. M.p. 156–158; Yield 88 percent; Proton NMR (300 MHz, DMSO D_6_): δ = 6.95–6.97 (Doublet, aromatic, 1H), 7.24–7.28 (multiplet, aromatic, 3H), 7.48–7.50 (Doublet, aromatic, 1H), 7.57–7.59 (Doublet, aromatic, 2H), 7.72 (Singlet, 1H, N-attached aryl hydrogen), 4.65 (s, 1H, dihydropyridine), 4.20 (s, 2H, NH_2_), 3.89 (s, OCH_3_, 3H), 3.62 (s, OCH_3_, 3H), 3.58 (s, OCH_3_, 3H) ppm; Carbon NMR in DMSO-*D_6_* (75 MHz): δ = 167.5 (Carbonyl) 161.2 (Carbonyl), 159.1 (Amine ‘C’), 148.3 (Ring N attached ‘C’), 142.1, 140.5, 139.7, 137.8, 135.2, 133.1, 130.4, 129.2, 126.4, 124.1, 122.7, 120.5, 117.9, 114.7 (CN), 64.2 (Methoxy), 60.1 (Methoxy), 58.8 (CN attached ‘C’), 55.1 (Methoxy), 35.4 (CH), ppm. Molecular formula = C_23_H_21_N_4_O_8_[(M + H)^+^]: HRMS m/z = Analyzed (481.1359)/Identified (481.1356).

### 3.2. Protein-Structure Preparation

Six-receptor-protein X-ray crystal structure, *Bacillus subtilis* (PDB ID: 6UF6) [33], *Staphylococcus aureus* (PDB ID: 6WN9) [34], *Escherichia coli* (PDB ID: 1HNJ) [35], *Proteus Vulgaris* (PDB ID: 5HXW) [36], *Aspergillus Niger* (PDB ID: 1UKC) [37], and *Aspergillus Flavus* (PDB ID: 5ZZD) [38] used the PDB (Protein Data Bank) in order to be retrieved [39]. PDB structures were altered via the protein-preparation wizard from the Schrödinger suite [40]. Prime restored the structural integrity of the protein-receptor residues by locating the missing side-chain atoms. After destroying the water molecules’ co-factors and ions, the hydrogen elements were subsequently recovered. Protonation and the condition of the tautomer altered at pH 3.0 [41].

#### Ligand-Structure Preparation

Avogadro software was implemented to draw and optimize a collection of 12 molecules of ligand (named 5a–l) [42,43]. For ligand generation and three-dimensional structure transformation, the optimized ligand sets were sent to LigPrep (New York-3, LLC, Schrdinger, and LigPrep). To regulate the shapes in .mae (Maestro) layout, the standard configurations for the OPLS3e force field were employed.

### 3.3. Antimicrobial Experiment

Drugs like ketoconazole and ciprofloxacin were used as references in investigations on antibacterial and antifungal effects. These medications were tested for solubility in 70% buffered propanol solvent. In order to prepare the test samples, dimethylformamide (DMF) was chosen as the solvent and DMSO-liquefied composites were used at a concentration of 200 μg/mL. 

#### 3.3.1. Antibacterial Screening Test

Two different groups of Gram-positive and Gram-negative pathogens mentioned in Section 2.3.1 became accustomed to screening the assay and used the method using a disc of filter paper [44]. The fundamental medium for evaluating bacteria was nutrient agar (NA). Only DMSO reagent was used for the disc controller. The agar Petri dishes were seeded with 0.5 mL of previously organized microbial suspension at 1 × 10^7^ cells mL^–1^. The dishes were incubated for 12 h at 37 °C for microbes and 24 h at 5 °C for diffusion time. The diameter of the visible area of inhibition was measured on a millimeter scale as a measure of the inhibition effect.

#### 3.3.2. Antifungal Screening

The test was performed using the poisoned diet procedure [45] with few adjustments and estimated for two plant pathogens and fungi, namely MTCC 1884 and MTCC 1881. The fungi were tested on a base medium called potato Dextrose agar (PDA). The further procedure followed the method cited in [26].

#### 3.3.3. MIC and MBC/MFC Effectiveness

The broth-dilution method was used for MIC screening [46]. In this method, freshly prepared food broth was used as the diluent. The earlier microorganism and fungal cultures (one day old) were mixed 100-fold in dietary broth (100 µL cultures of bacterial in 10 mL of NB). The experimental tubes containing the provided bacterial and fungal cultures received increasing amounts of the test samples (40, 20, 10, 5, 205, and 1.25 µL of stock solution holds 100, 50, 25, 12.5 and 6.25 µg of the test motifs). In addition, these portions underwent cultivation for fungus and germs, respectively, at 28 °C for 48 h and 37 °C for 1 day. The solvent control and discernible turbidity of each part were checked using an NB regulator, and no test-sample controls were tested simultaneously. The MIC was measured as the minimum concentration that prevented visible progression of the organisms tested, using each test tube set in the MIC determination to screen for MBC [47] and MFC [48]. From the tubes showing no growth, a loop of the broth was removed and inoculated by streaking with sterile nutrient broth against microbes and PDA against fungi. The portions hatched at 37 °C for one day after being injected with bacteria and inoculated with fungi, followed by culturing at 28 °C for 48 h. The MBC or MFC has been restricted as the lowest concentration of the test samples intended to kill microbes or fungi.

### 3.4. Cytotoxic Assessment

#### 3.4.1. Cell Lines and Cell Culture

The human histiocytic carcinoma U937, SKOV3 (human ovarian carcinoma), HepG2 (human hepatocellular carcinoma) and B16F10 (mouse melanoma) through the NCCS (National Centre for Cellular Sciences), India- Pune, cell lines were gathered. The appropriate selection media were used to cultivate the cells (U937 (1640-RPMI), MEM (HepG2), and DMEM (SKOV3, B16F10)), and 10% FBS (fetal bovine serum) that has been heat inactivated was added. A total of 100 Units/mL of penicillin, 2 mM of glutamine, 1 mM of sodium bicarbonate, and 100 g/mL of streptomycin were all used. At 37 degrees Celsius, in a humidified atmosphere with 5% CO_2_, all cell lines were cultivated.

#### 3.4.2. Concentrations Tested

Before the commencement of every study, fresh stock solutions of each chemical and a positive control were made. Each chemical was produced as a stock solution at an 8 mg/mL concentration in 100% DMSO. By successive dilutions with the suitable medium for cultivation, functioning solutions of every experiment chemical were created. All substances underwent testing at the necessary concentrations ranging from 10 to 200 µg/mL.

#### 3.4.3. Cytotoxicity

Following the Mossman protocol [49], the MTT assay [3-(4,5-dimethylthiazol-2-yl)-2,5-diphenyltetrazolium bromide] was used to quantify the cytotoxicity. In 96-well plates, cells (2x104) were cultivated into every well with 100 µL of medium. An exact 100 μL of medium with various experimental doses (10 g to 200 μg/mL) was injected to the cell culture after incubation for an overnight period (at 37 °C and in an atmosphere that was humidified with 5% carbon dioxide). This equates to between 2 and 40 μg per 200 μL test quantity. After one day, the viability of cells was determined via incorporating 10 µL of MTT (5 mg/mL), each well and continuing the incubation for an additional 3 h at 37 °C. The formazan blue that had developed in the tissues disappeared in 100 μL of DMSO after the medium had been removed. At 570 nm, a spectrophotometer (MAX Plus Spectra; SOFTmax PRO-5.4 supported molecular device) was used to measure the brightness of color production. The proportion of viable cells that were inhibited was calculated in comparison to control (no test substance) readings. Mean values were evaluated using regression analysis to find the finest straight-line agreement after collecting data from three different experiments. Using appropriate regression algorithms, IC50 concentrations (resulting in a 50% reduction in cell count compared to control measurements) were determined.

## 4. Conclusions

An environmental favor approach was established for the synthesis of novel 1,4-dihydropyridine substances. There was good yield in the formation of whole compounds. Also, docking studies were performed for targets with four bacterial and two fungal pathogens, in which compound **5f** showed a higher binding energy versus four microbes. In fact, other dihydropyridines and their docking scores against each pathogen have been widely discussed. Besides, the inhibitory activity of the compound against microbes was examined. Among other things, compound **5f** was responsible for highly antimicrobial activity. Moreover, MIC and MBC/MFC data showed that **5f** had a minimal bacterial concentration versus the *E. coli* bacterium. Also, whole motifs were tested for their cytotoxic activity. It was found that the methoxy- and hydroxy-linked compounds (**5h**) showed greater cytotoxic potency, followed by the two hydroxy-linked compounds (**5d** and **5f**). A description of the biological investigation results are as follows: DHP analogs serve as fertile ground for the next generation of innovative antibacterial/cytotoxic drugs.

## Figures and Tables

**Table 1:** Different strategies and yields of compound (**5a–c**).

Entry No	Initiator	Solvent	Yield ^a^
1	PEG-400 ^b^	-	5a (45); 5b (39), 5c (41)
2	ZnCl_2_	Cyanomethane	5a (22); 5b (15), 5c (29)
3	InCl_3_	Cyanomethane	5a (11); 5b (19), 5c (15)
4	SnCl_2._H_2_O	Cyanomethane	5a (32); 5b (25), 5c (21)
5	Clay acid *	Ethanol–water	5a (86); 5b (83), 5c (88)

^a^ Procedure for 1–4 entries: Alkyne **2** (1 mmol) was injected into a combination of related aryl aldehyde **3** (1 mmol), malononitrile (**4**) (1 mmol) and respective aryl amine **1** (1 mmol) corresponding catalyst (1.5 mmol) in acetonitrile (12 mL) reflux for 5–8 h at 80 °C. followed by TLC and column to obtain corresponding products **5a**, **5b** and **5c**. * Montmorillonite K-10 acid clay; ^b^ all respective initial compounds of products in PEG-400 and refluxed followed by purification.

**Table 2:** Binding energy and molecular interactions of *Bacillus subtilis* with twelve ligands.

Compound ID	Glide Score	Glide Emodel	Hydrogen Bond	Hydrophobic Interaction	Polar Interaction	Pi–Cation Interaction	Pi–Pi Stacking Interaction	Negative Interaction	Positive Interaction	Glycine Interaction
*Bacillus subtilis* (PDB: 6UF6)										
**5a**	−3.593	−32.404	Arg205, Asp212	Ile88, Val102, Phe172, Phe208	Ser103, Asn169, Thr171, Gln216, Gln270	--	Phe208	Asp212, Glu272	Mse101, Arg205, Arg215, Hip271	--
**5b**	−3.004	−31.562	Asp212, Gln216, Hip271	Phe208, Ile268	Asn169, Thr171, Gln216, Gln270	--	--	Asp212, Glu272	Arg215, Hip271	--
**5c**	−2.805	−32.360	Asp212, Gln216, Gln270	Phe208, Ile268	Asn169, Thr171, Gln216, Gln270	--	--	Asp212, Glu272	Arg205, Arg215, Hip271	--
**5d**	−3.420	−28.754	Asn169, Gln216, Gln270	Phe208, Ile268	Asn169, Thr171, Gln216, Gln270	--	--	Asp212, Glu272	Arg205, Arg215, Lys269, Hip271	--
**5e**	−2.677	−24.648	Arg205, Gln270	Val102, Phe208, Ile268	Ser103, Asn169, Thr171, Gln270	--	Phe208	Asp212, Glu272	Arg205, Arg215, Lys269, Hip271	--
**5f**	−3.951	−29.177	Ser103, Arg205, Gln270, Hip271	Val102, Phe208, Ile268	Ser103, Asn169, Thr171, Gln270	--	Phe208	Asp212, Glu272	Arg205, Arg215, Lys269, Hip271	--
**5g**	−2.978	−26.916	Arg205, Gln270	Phe208, Ile268	Ser103, Asn169, Thr171, Gln270	--	Phe208	Asp212, Glu272	Arg205, Arg215, Lhip271	--
**5h**	−3.211	−31.744	Arg205, Asp212, Gln270	Phe172, Pro204, Phe208, Ile268	Asn169, Thr171, Gln216, Ser259, Gln270	--	--	Asp212	Arg205, Arg215	--
**5i**	−3.103	−36.652	Arg205, Arg215, Gln216, Gln270	Phe172, Phe208, Ile268	Asn169, Thr171, Gln216, Ser259, Gln270	--	--	Asp212	Arg205, Arg215	--
**5j**	−2.945	−34.957	Arg215, Gln216, Gln270	Phe172, Phe208, Ile268	Asn169, Thr171, Gln216, Ser259, Gln270	--	--	Asp212	Arg205, Arg215	--
**5k**	−3.213	−39.572	Glu185	Val168, Ile257, Ile268	Thr99, Asn169, Gln216, Gln219	--	--	Glu185	Arg215	--
**5l**	−2.896	−34.358	Arg205, Asp212, Gln270	Phe172, Phe208, Ile268	Asn169, Thr171, Gln216, Ser259, Gln270	--	--	Asp212	Arg205, Arg215	--

**Table 3:** Binding energy and molecular interactions of *Staphylococcus aureus* with twelve ligands.

Compound ID	Glide Score	Glide E-Model	Hydrogen Bond	Hydrophobic Interaction	Polar Interaction	Pi–Cation Interaction	Pi–Pi Stacking Interaction	Negative Interaction	Positive Interaction	Glycine Interaction
*Staphylococcus aureus* (PDB: 6WN9)										
**5a**	−3.910	−31.859	Ser445, Lys499	Pro447, Phe462, Ile466, Pro467	Ser445, Asn468	--	--	Glu596	Lys499	--
**5b**	−3.525	−38.417	Asn468	Pro447, Ile466,Pro467, Val592, Met595	Ser445, Asn468, Gln498	--	--	Glu596	Lys499	--
**5c**	−3.291	−39.247	Gly497	Pro447, Ile466, Val592, Met595	Ser445, Gln498	--	--	Glu596	Lys499	Gly497
**5d**	−3.299	−38.468	Ser445, Gly497	Pro447, Ile466 Val592, Met595	Ser445, Gln498, Hie598	Lys499	--	Glu596	Lys499	Gly497
**5e**	−3.269	−36.366	Gly497	Pro447, Phe462, Ile466, Val592, Met595	Ser445, Ser446, Asn468, Gln498	--	--	Glu596	Lys499	Gly497
**5f**	−3.916	−44.854	Ser445, Asn468, Lys499,	Pro447, Phe462, Ile466, Pro467, Val592, Met595	Ser445, Asn468, Gln498, Hie598	Lys499	--	Glu596	Lys465, Lys499	--
**5g**	−3.393	−35.834	Ser445, Gly497	Pro447, Phe462, Ile466, Val592, Met595	Ser445, Asn468, Gln498	Lys499	--	Glu596	Lys496, Lys499	Gly497
**5h**	−3.584	−33.437	Ser445, Gly497	Pro447, Phe462, Ile466, Val592, Met595	Ser445, Asn468, Gln498	Lys499	--	Glu596	Lys496, Lys499	Gly497
**5I**	−3.084	−35.310	Gly497	Pro447, Ile466, Val592, Met595	Ser445, Gln498, Hie598	Lys499	--	Glu596	Lys499	Gly497
**5j**	−3.919	−36.527	Lys499	Pro447, Phe462, Ile466, Pro467, Val592, Met595	Ser445, Asn468,	--	--	Glu596	Lys499	--
**5k**	−3.619	−38.594	Asn468	Pro447, Ile466, Pro467, Val592, Met595	Ser445, Ser446, Asn468, Gln498	--	--	Glu596	Lys499	Gly497
**5l**	−3.760	−36.242	Ser445, Gly497	Pro447, Phe462, Ile466, Val592, Met595	Ser445, Asn468, Gln498	Lys499	--	Glu596	Lys499	Gly497

**Table 4:** Binding energy and molecular interactions of *Escherichia coli* with twelve ligands.

Compound ID	Glide Score	Glide E-Model	Hydrogen Bond	Hydrophobic Interaction	Polar Interaction	Pi–Cation Interaction	Pi–Pi Stacking Interaction	Negative Interaction	Positive Interaction	Glycine Interaction
*Escherichia coli* (PDB: 1HNJ)										
**5a**	−5.007	−38.227	Asn210 (2)	Trp32, Ile155, Ile156, Met207,Val212, Phe213, Ala246, Ile250	Thr37, Thr153, Thr206, Asn210, Asn247	Arg36	--	Asp150	Arg36, Arg249	Gly152, Gly209,
**5b**	−4.810	−48.379	Asn247 (2)	Trp32, Ile155, Ile156, Met207, Phe213, Ala246	Thr28, Thr37, Asn210, Asn247	Arg36,	Trp32	Asp27	Arg36, Arg151, Arg249	Gly152, Gly209,
**5c**	−4.797	−40.220	Arg36, Gly209, Gly152	Trp32, Ile156, Phe157, Met207, Val212, Phe213, Ala246, Ile250	Thr37, Asn210, Asn247, Asn274	--	--	--	Arg36, Arg249	Gly152, Gly209,
**5d**	−4.570	−40.380	Arg36, Gly209	Trp32, Ile155, Ile156,Met207, Phe213, Ala246	Thr37, Asn210, Asn247	Arg36,	Trp32	--	Arg36, Arg151, Arg249	Gly152, Gly209,
**5e**	−5.119	−47.951	Asn247 (2)	Trp32, Ile155, Ile156,Met207, Phe213, Ala246	Thr28, Thr37, Asn210, Asn247	--	Trp32	Asp27	Arg36, Arg151	Gly152, Gly209,
**5f**	−4981	−44.531	Asn247 (2)	Trp32, Ile155, Ile156, Met207, Phe213, Ala246	Thr28, Thr37, Asn210, Asn247	--	Trp32	Asp27	Arg36, Arg151	Gly152, Gly209,
**5g**	−5.099	−46.773	Asn247 (2)	Trp32, Ile155, Ile156, Met207, Phe213, Ala246,	Thr28, Thr37, Asn210, Asn247	--	Trp32	Asp27	Arg36, Arg151,	Gly152, Gly209,
**5h**	−5.237	−42.775	Asn210	Trp32, Ile155, Ile156, Met207, Val212, Phe213, Ala246, Ile250	Thr37, Thr153, Asn210, Asn247	Arg36,	--	Asp150	Arg36, Arg249	Gly152, Gly209,
**5i**	−5.012	−38.890	Met207, Asn210 (2)	Trp32, Ile155, Ile156, Met207, Ala208, Phe213, Ala246, Ile250	Thr37, Thr153, Asn210, Asn247	Arg36,	--	--	Arg36, Arg249	Gly152, Gly209,
**5j**	−4.753	−35.678	Asn210 (2)	Trp32, Ile155, Ile156, Met207, Phe213, Ala246, Ile250	Thr37, Thr153, Asn210, Asn247	Arg36,	--	Asp150	Arg36, Arg249	Gly152, Gly209,
**5k**	−5.414	−46.281	Asn247 (2)	Trp32, Ile155, Ile156, Met207, Phe213, Ala246	Thr28, Ser29, Thr37, Asn210, Asn247	--	Trp32	Asp27	Arg36, Arg151	Gly152, Gly209,
**5l**	−5.900	−49.830	Asn210 (2)	Trp32, Ile155, Ile156, Met207, Val212, Phe213,Ala246, Ile250	Thr37, Thr153, Thr206, Asn210, Asn247	Arg36,	--	Asp150	Arg36, Arg249	Gly152, Gly209,

**Table 5:** Binding energy and molecular interactions of *Proteus vulgaris* with twelve ligands.

Compound ID	Glide Score	Glide E-Model	Hydrogen Bond	Hydrophobic Interaction	Polar Interaction	Pi–Cation Interaction	Pi–Pi Stacking Interaction	Negative Interaction	Positive Interaction	Glycine Interaction
*Proteus vulgaris* (PDB: 5HXW)										
**5a**	−8.235	−58.597	Gln92, Gly98, Gln99	Ile64, Leu65, Phe96, Tyr97, Phe201, Ile317, Trp407, Ala410, Met411, Ile413, Trp438, Met440	Gln92, Ser93, Gln99, Gln278, Gln280, Thr436, Thr441	--	--	Glu91	Arg95	Gly98, Gly257, Gly409, Gly437, Gly439
**5b**	−7.815	−48.721	Gln92, Gly98, Gln99, Gln278	Ile64, Leu65, Phe96, Tyr97, Phe201, Ile317, Ala410, Met411, Trp438, Met440	Gln92, Ser93, Gln99, Gln278, Gln280, Ser313, Thr436, Thr441	Tyr97	--	--	Arg95, Arg315	Gly98, Gly257, Gly409, Gly437, Gly439
**5c**	−7.076	−44.009	Gln92, Tyr97, Gly98, Gln99, Thr436	Ile64, Leu65, Phe96, Tyr97, Phe201 Ile317, Ala410, Met411, Trp438, Met440	Gln92, Ser93, Gln99, Gln278, Gln280, Ser313, Thr436, Thr441	--	Trp438	--	Arg95	Gly63, Gly98, Gly409, Gly437, Gly439
**5d**	−7.888	−49.092	Thr336, Met440,	Ile64, Leu65, Phe96, Tyr97, Ala100, Phe201, Ile317, Met411, Trp438, Met440	Gln92, Ser93, Gln99, Gln278, Gln280, Ser313, Thr436, Thr441	--	--	Glu442	Arg95	Gly98, Gly409, Gly437, Gly439
**5e**	−7.218	−62.271	Gly98, Gln99	Ile64, Leu65, Phe96, Tyr97, Phe201, Trp407, Ala410, Met411, Trp438, Met440	Gln92, Ser93, Gln99, Gln278, Gln280, Thr436, Thr441	--	--	--	Arg95	Gly98, Gly257, Gly409, Gly437, Gly439
**5f**	−8.499	−55.594	Gln92, Gly98, Gln99	Ile64, Leu65, Phe96, Tyr97, Phe201, Ile317, Trp407, Ala410, Met411, Trp438, Met440	Gln92, Ser93, Gln99, Gln278, Gln280, Thr436, Thr441	--	Trp438	Glu91	Arg95	Gly98, Gly409, Gly437, Gly439
**5g**	−7.163	−60.285	Gly98, Gln99	Ile64, Leu65, Phe96, Tyr97, Phe201 Trp407, Ala410, Met411, Trp438, Met440	Gln92, Ser93, Gln99, Gln278, Gln280, Thr436, Thr441	--	--	--	Arg95	Gly98, Gly257, Gly409, Gly437, Gly439
**5h**	−7.733	−38.497	Gln92, Gly98, Gln99, Trp438	Ile64, Leu65, Phe96, Tyr97, Phe201, Phe301, Ile317, Ala410, Met411, Trp438, Met440	Gln92, Ser93, Gln99, Gln278, Gln280, Ser313	--	Trp438	--	Arg95	Gly63, Gly98, Gly409, Gly437, Gly439
**5i**	−7.213	−59.216	Gly98, Gln99	Ile64, Leu65, Phe96, Tyr97, Phe201, Trp407, Ala410, Met411, Ile413, Trp438, Met440	Gln92, Ser93, Gln99, Gln278, Gln280, Thr436, Thr441	--	--	--	Arg95	Gly98, Gly257, Gly409, Gly437, Gly439
**5j**	−8.053	−60.362	Gln92, Gly98, Gln99	Ile64, Leu65, Phe96, Tyr97, Phe201, Trp407, Ala410, Met411, Ile413, Trp438, Met440	Gln92, Ser93, Gln99, Gln278, Gln280, Thr436, Thr441	--	--	Glu91	Arg95	Gly63, Gly98, Gly409, Gly437, Gly439
**5k**	−8.322	−60.500	Gln92, Tyr97, Gly98, Gln99	Ile64, Leu65, Phe96, Tyr97, Phe201 Ile317, Trp407, Ala410, Met411, Trp438, Met440	Gln92, Ser93, Gln99, Gln278, Gln280, Thr436, Thr441	--	--	Glu91	Arg95	Gly98, Gly257, Gly409, Gly437, Gly439
**5l**	−7.866	−63.768	Gly98, Gln99, Thr436	Ile64, Leu65, Phe96, Tyr97, Phe201, Trp407, Ala410, Met411, Trp438, Met440	Gln92, Ser93, Gln99, Gln278, Gln280, Thr436, Thr441	--	--	--	Arg95	Gly63, Gly98, Gly409, Gly437, Gly439

**Table 6:** Binding energy and molecular interactions of *Aspergillus niger* with twelve ligands.

Compound ID	Glide Score	Glide E-Model	Hydrogen Bond	Hydrophobic Interaction	Polar Interaction	Pi–Cation Interaction	Pi–Pi Stacking Interaction	Negative Interaction	Positive Interaction	Glycine Interaction
*Aspergillus niger* (PDB: 1UKC)										
**5a**	−6.731	−64.828	Asn134, Asn341	Tyr137, Val209, Trp301, Phe342, Ile436, Pro439, Phe442, Leu444, Pro445, Leu456, Tyr462	Ser133, Asn134, Ser210, Asn341, Asn431, His440, Thr441, Thr453, Ser460	--	--	--	--	Gly126, Gly127, Gly128
**5b**	−6.828	−70.326	Asn134, Asn341	Tyr137, Val209, Trp301, Phe342, Ile436, Pro439, Phe442, Leu444, Pro445, Leu456, Tyr462	Ser133, Asn134, Ser210, Asn341,Asn431, His440, Thr441, Thr453, Ser460	--	--	--	--	Gly126, Gly127, Gly128
**5c**	−4.982	−45.227	Asn134, Thr441, Thr453	Tyr137, Val209, Phe342, Pro439, Phe442, Leu444, Pro445, Leu456, Tyr462	Ser133, Asn134, Ser210, Asn341, Asn431, His440, Thr441, Thr453, Thr455, Ser457, Ser460	--	--	--	--	Gly127, Gly454
**5d**	−6.632	−66.774	Asn134, Asn341	Tyr137, Val209, Phe342, Ile436, Pro439, Phe442, Leu444,Pro445, Leu456, Tyr462	Ser133, Asn134, Ser210, Asn341, Asn431, His440, Thr441, Thr453	--	His440	--	--	Gly126, Gly127, Gly128
**5e**	−3.751	−44.902	--	Ala211,Trp301, Phe342,Ile436, Pro439,Phe442, Leu444,Pro445	Ser133, Asn134, Ser210, Asn341,His440, Thr441	--	--	--	--	Gly126, Gly127, Gly128
**5f**	−6.895	−67.799	Asn134, Asn341	Tyr137, Val209, Trp301, Phe342, Ile436, Pro439, Phe442, Leu444, Pro445, Leu456, Tyr462	Ser133, Asn134, Ser210, Asn341, Asn431, His440, Thr441, Thr453, Ser460	--	--	--	--	Gly126, Gly127, Gly128
**5g**	−6.703	−68.366	Ser522	Tyr375, Leu381, Ala393, Val518, Pro519	Thr336, Ser392, Thr401, Ser522	Arg423	--	Ash334, Ash335, Asp337, Asp397, Asp525	Arg423, Arg505	Gly389
**5h**	−3.333	−46.158	Ser522, Arg423	Tyr375, Leu381, Phe388, Ala393, Val518, Pro519	Thr336, Ser340, Ser392, Thr401, Ser522	Arg423	--	Ash334, Ash335, Asp337, Asp525	Arg423, Arg505	Gly389
**5i**	−4.785	−45.484	His440	Tyr137, Trp301, Phe342, Ile436, Pro439, Phe442, Leu444, Pro445, Leu456, Tyr462	Ser133, Asn134, Ser210, Asn341,Asn431, His440, Thr441, Thr453, Ser460	--	--	--	--	Gly127, Gly454
**5j**	−5.873	−58.046	Ser210, Asn341	Tyr137, Ala211, Trp301, Phe342, Ile436, Pro439, Phe442, Leu444, Pro445, Leu456, Tyr462	Ser133, Asn134, Ser210, Asn341,Asn431, His440, Thr441, Thr453, Ser460	--	--	Glh131	--	Gly126, Gly127, Gly128
**5k**	−6.435	−64.546	Asn134, Asn341	Tyr137, Val209, Trp301, Phe342, Ile436, Pro439, Phe442, Leu444, Pro445, Leu456, Tyr462	Ser133, Asn134, Ser210, Asn341,Asn431, His440, Thr441, Thr453, Ser460	--	--	--	--	Gly126, Gly127, Gly128
**5l**	−3.167	−46.817	Ser522	Tyr375,Leu381,Ala393, Val518, Pro519	Thr336, Ser392, Ser522	Arg423	--	Ash334, Ash335, Asp337, Asp397, Asp525	Arg423, Arg505	Gly389

**Table 7:** Binding energy and molecular interactions of *Aspergillus flavus* with twelve ligands.

Compound ID	Glide Score	Glide E-Model	Hydrogen Bond	Hydrophobic Interaction	Polar Interaction	Pi–Cation Interaction	Pi–Pi Stacking Interaction	Negative Interaction	Positive Interaction	Glycine Interaction	Salt Bridge
*Aspergillus flavus* (PDB: 5ZZD)											
**5a**	−5.415	−66.838	Gly227, Asp296	Phe176, Leu192, Leu193, Met230, Leu292, Ile293	Hie232	--	--	Asp195, Glu196, Asp296	Arg197, Arg291	Gly227, Gly228, Gly229	--
**5b**	−5.000	−66.164	Gly227	Phe176, Phe189, Leu192, Leu193, Leu253, Val256, Phe276, Leu292, Ile293	--	--	--	Asp195, Glu196, Asp252, Asp296	Arg197, Arg291, Arg295	Gly227, Gly228,	Asp195
**5c**	−5.230	−65.947	Gly227, Gly228, Arg291	Phe176, Phe189, Leu192, Leu193, Met230, Val256, Phe276, Leu292, Ile293	Hie232	--	--	Asp195, Glu196, Asp296	Arg197, Arg291	Gly227, Gly228, Gly229, Gly231	--
**5d**	−5.902	−68.806	Gly227, Gly228, Arg291	Phe176, Phe189, Leu192, Leu193, Met230, Phe276, leu292, Ile293	Hie232	--	--	Asp195, Glu196, Asp296	Arg197, Arg291	Gly227, Gly228, Gly229, Gly231	--
**5e**	−4.885	−63.587	Gly227, Asp296	Phe176, Phe189, Leu192, Leu193, Leu292, Ile293	Hie232	--	--	Asp195, Glu196, Asp296	Arg197, Arg291	Gly227, Gly228, Gly229	--
**5f**	−5.991	−72.025	Arg197, Gly227, Asp296	Phe176, Phe189, Leu192, Leu193, Leu292, Ile293	Hie232	--	--	Asp195, Glu196, Asp296	Arg197, Arg291	Gly227, Gly228, Gly229	--
**5g**	−5.362	−63.817	Gly227, Asp296	Phe176, Phe189, Leu192, Leu193, Leu292, Ile293	Hie232	--	--	Asp195, Glu196, Asp296	Arg197, Arg291	Gly227, Gly228, Gly229	--
**5h**	−5.980	−69.543	Gly227, Asp296	Phe176, Phe189, Leu192, Leu193, Leu253, Leu292, Ile293	Asn200, Hie232	--	--	Asp195, Glu196, Asp296	Arg197, Arg291	Gly227, Gly228, Gly229	--
**5i**	−5.032	−63.403	Gly227, Asp296	Phe176, Phe189, Leu192, Leu193, Leu292, Ile293	Hie232	--	--	Asp195, Glu196, Asp296	Arg197, Arg291	Gly227, Gly228, Gly229	--
**5j**	−5.249	−64.825	Gly227, Asp296	Phe176, Phe189, Leu192, Leu193, Leu292, Ile293	Hie232	--	--	Asp195, Glu196, Asp296	Arg197, Arg291	Gly227, Gly228, Gly229	--
**5k**	−4.893	−64.395	Gly227	Phe176, Phe189, Leu192, Leu193, Leu292, Ile293	--	--	--	Asp195, Glu196, Asp252, Asp296	Arg197, Arg291, Arg295	Gly227, Gly229,	--
**5l**	−5.250	−62.550	Arg197, Gly227, Asp296	Phe176, Phe189, Leu192, Leu193, Leu253, Leu292, Ile293	Hie232	--	--	Asp195, Glu196, Asp252, Asp296	Arg197, Arg291, Arg295	Gly227, Gly228, Gly229	--

**Table 8:** The in vitro pathogenic potential of targets **5(a–l)**.

Compound No.	Zone of Inhibition for Antimicrobials (mm) after 1 Day at 200 g/mL Concentration					
	Bacterial and Its Resistance				Fungal and Its Resistance	
	Gram-Positive		Gram-Negative			
	*Bacillus subtilis*	*Staphylococcus aureus*	*Escherichia coli*	*Proteus vulgaris*	*Aspergillus niger*	*A. flavus*
**5a**	35.21	39.19	30.05	34.21	32.31	36.22
**5b**	30.31	33.36	24.42	27.29	25.15	29.32
**5c**	19.14	21.19	14.08	15.21	13.29	17.09
**5d**	10.21	11.31	06.29	08.05	07.32	09.20
**5e**	16.05	18.07	10.01	14.16	10.41	12.16
**5f**	40.12	42.14	38.05	39.22	36.19	40.14
**5g**	07.18	09.23	04.11	05.31	06.02	08.21
**5h**	22.41	28.12	16.15	19.42	18.30	24.05
**5i**	05.32	06.10	02.16	04.01	04.38	05.11
**5j**	24.02	29.05	20.06	21.33.	22.22	26.11
**5k**	38.15	41.03	35.11	37.16	35.03	39.05
**5l**	34.05	36.30	25.21	29.07	30.42	33.17
Ciprofloxacin	41.22	44.30	36.21	40.22	-	-
Ketoconazole	-	-	-	-	38.45	41.24
Control(DMSO)	-	-	-	-	-	-

**Table 9:** MFC, MIC and MBC of substances **5a**, **5b**, **5f**, **5k**, and **5l**.

Compound No.	MIC					
	MIC (MBC/MFC) μg/mL					
	*B. subtilis*	*S. aureus*	*E. coli*	*P. vulgaris*	*A. niger*	*A. flavus*
**5a**	25 (50)	12.5 (100)	25 (100)	50 (200)	12.5 (50)	50 (100)
**5b**	50 (200)	25 (>200)	50 (100)	50 (200)	12.5 (25)	12.5 (100)
**5f**	25 (100)	12.5 (25)	50 (>200)	50 (200)	25 (50)	50 (100)
**5k**	50 (200)	100 (>200)	12.5 (25)	25 (100)	50(>200)	12.5 (25)
**5l**	12.5 (200)	50 (100)	25 (50)	50 (>200)	25 (100)	12.5 (100)
Ciprofloxacin	12.5	6.25	12.5	12.5	-	-
Ketoconazole	-	-	-	-	12.5	6.25

**Table 10:** Compounds **5(a–l)** cytotoxic potency values by using MTT assay against HepG-2, U937 and SKOV3 cell lines.

Tested Target	IC50 (μg/mL)		
	HepG-2	U937	SKOV3
Etoposide	2.02 ± 0.15	5.23 ± 3.01	6.81 ± 1.56
**5a**	65.16 ± 1.45	101.49 ± 1.09	119.45 ± 1.35
**5b**	86.32 ± 1.05	123.01 ± 1.65	160.23 ± 1.41
**5c**	128.71 ± 1.30	>200	>200
**5d**	57.45 ± 0.95	74.20 ± 2.01	88.14 ± 2.20
**5e**	147.26 ± 1.01	168.71 ± 2.11	>200
**5f**	42.11 ± 2.11	60.12 ± 2.61	75.43 ± 0.99
**5g**	71.94 ± 0.54	>200	>200
**5h**	29.56 ± 1.20	36.05 ± 1.00	51.25 ± 0.22
**5i**	112.55 ± 2.46	131.33 ± 1.05	>200
**5j**	84.46 ± 0.89	117.46 ± 1.19	129.50 ± 2.30
**5k**	59.78 ± 1.12	83.12 ± 3.46	95.09 ± 2.14
**5l**	80.28 ± 3.01	110.51 ± 0.51	124.13 ± 1.02

## Data Availability

Data is contained within the article.

## References

[1] Kappe C.O., Fabian W.M., Semones M.A. (1997). Conformational analysis of 4-aryl-dihydropyrimidine calcium channel modulators. A comparison of ab initio, semiempirical and X-ray crystallographic studies. Tetrahedron.

[2] Rovnyak G.C., Kimball S.D., Beyer B., Cucinotta G., DiMarco J.D., Gougoutas J., Hedberg A., Malley M., McCarthy J.P. (1995). Calcium entry blockers and activators: Conformational and structural determinants of dihydropyrimidine calcium channel modulators. J. Med. Chem..

[3] Hilgeroth A. (2022). Dimeric 4-Aryl-1, 4-dihydropyridines: Development of a third class of nonpeptidic HIV-1 protease inhibitors. Mini-Rev. Med. Chem..

[4] Nair V., Offerman R.J., Turner G.A. (1986). Novel fluorescent 1,4-dihydropyridines. J. Am. Chem. Soc..

[5] Gomha S.M., Edrees M.M., Muhammad Z.A., Kheder N.A., Abu-Melha S., Saad A.M. (2020). Synthesis, characterization, and antimicrobial evaluation of some new 1, 4-dihydropyridines-1, 2, 4-triazole hybrid compounds. Polycycl. Aromat. Compd..

[6] Pan C., Luo M., Lu Y., Pan X., Chen X., Ding L., Che J., He Q., Dong X. (2022). Design, synthesis and biological evaluation of new dihydropyridine derivatives as PD-L1 degraders for enhancing antitumor immunity. Bioorg. Chem..

[7] Cabrera D.C., Santa-Helena E., Leal H.P., de Moura R.R., Nery L.E.M., Gonçalves C.A.N., Russowsky D., D‘Oca M.G.M. (2019). Synthesis and antioxidant activity of new lipophilic dihydropyridines. Bioorg. Chem..

[8] Sobolev A., Franssen M.C.R., Duburs G., de Groot A.E. (2004). Chemoenzymatic synthesis of enantiopure 1, 4-dihydropyridine derivatives. Biocatal. Biotransform..

[9] Marchalín S., Chudík M., Mastihuba V., Decroix B. (1998). Use of enzymes in preparation of enantiopure 1, 4-dihydropyridines. Heterocycles.

[10] Sadeghzadeh S.M. (2016). Bis(4-pyridylamino) triazine-stabilised magnetite KCC-1: A chemoselective, efficient, green and reusable nanocatalyst for the synthesis of Nsubstituted 1,4-dihydropyridines. RSC Adv..

[11] Amoozadeh A., Rahmani S., Bitaraf M., Abadi F.B., Tabrizian E. (2016). Nano-zirconia as an excellent nano support for immobilisation of sulfonic acid: A new, efficient and highly recyclable heterogeneous solid acid nanocatalyst for multicomponent reactions. New J. Chem..

[12] Hadebe N.P., Kerru N., Tukulula M., Jonnalagadda S.B. (2022). A sustainable molybdenum oxide loaded on zirconia (MoO_3_/ZrO_2_) catalysed multicomponent reaction to synthesis novel dihydropyridines. Sustain. Chem. Pharm..

[13] Anastas P.T. (1994). Benign by design chemistry. Benign by Design.

[14] Majee S., Shilpa, Sarav M., Banik B.K., Ray D. (2023). Recent Advances in the Green Synthesis of Active N-Heterocycles and Their Biological Activities. Pharmaceuticals.

[15] Tundo P., Anastas P.T. (2000). Green Chemistry, Challenging Perspectives.

[16] Anastas P.T., Warner J.C. (1998). Green Chemistry: Theory and Practice.

[17] Karadeniz B., Howarth A.J., Stolar T., Islamoglu T., Dejanovi’c I., Tireli M., Wasson M.C., Moon S.Y., Farha O.K., Friscic T. (2018). Benign by design: Green and scalable synthesis of zirconium UiO-metal-organic frameworks by water-assisted mechanochemistry. ACS Sustain. Chem. Eng..

[18] Al-Zaydi K.M., Al-Boqami M., Elnagdi N.M. (2022). Green Synthesis of Dihydropyrimidines and Pyridines Utilizing Biginelli Reaction. Polycycl. Aromat. Compd..

[19] Draye M., Chatel G., Duwald R. (2020). Ultrasound for Drug Synthesis: A Green Approach. Pharmaceuticals.

[20] Dige N.C., Pore D.M. (2015). Green Aspect for Multicomponent Synthesis of Spiro [4H-indeno [1,2-b] pyridine-4, 3′-[3H] indoles]. Synth. Commun..

[21] Giridhar R. (2012). Drug discovery: Past and present. J. Adv. Pharm. Technol. Res. JAPTR.

[22] Basha N.M., Venkatesh B.C., Reddy G.M., Zyryanov G.V., Subbaiah M.V., Wen J.-C., Gollakota A.R.K. (2023). Synthesis, Antimicrobial Assay and SARs of Pyrazole Included Heterocyclic Derivatives. Polycycl. Aromat. Compd..

[23] Karthick R., Velraj G., Karuppusamy A., Karthikeyan S. (2023). Experimental, Molecular Docking and Molecular Dynamics Investigation on Newly Synthesized Diethyl 4-(Anthracen-9-yl)-2, 6-Dimethyl-1, 4-Dihydropyridine-3, 5-Dicarboxylate. Polycycl. Aromat. Compd..

[24] Basha N.M., Seenaiah D., Padmaja A., Padmavathi V., Bhargav D.S., Vijaya T. (2014). Synthesis and antioxidant activity of Bis and Tris Heterocycles. Arch. Der Pharm..

[25] Veligeti R., Madhu R.B., Anireddy J., Pasupuleti V.R., Avula V.K.R., Ethiraj K.S., Uppalanchi S., Kasturi S., Perumal Y., Anantaraju H.S. (2020). Syn thesis of novel cytotoxic tetracyclic acridone derivatives and study of their molecular docking, AD MET, QSAR, bioactivity and protein binding properties. Sci. Rep..

[26] Gündüz M.G., Dengiz C., Aslan E.K., Bogojevic S.S., Nikodinovic-Runic J. (2022). Attaching azoles to Hantzsch 1, 4-dihydropyridines: Synthesis, theoretical investigation of nonlinear optical properties, antimicrobial evaluation and molecular docking studies. J. Mol. Struct..

[27] (2018). Glide.

[28] Dubey A., Marabotti A., Ramteke P.W., Facchiano A. (2016). In silico approach to find chymase in hibitors among biogenic compounds. Future Med. Chem..

[29] Dubey A., Marabotti A., Ramteke P.W., Facchiano A. (2016). Interaction of human chymase with gink golides, terpene trilactones of Ginkgo biloba investigated by molecular docking simulations. Biochem. Biophys. Res. Commun..

[30] Bharadwaj S., Dubey A., Kamboj N.K., Sahoo A.K., Kang S.G., Yadava U. (2021). Drug repurposing for ligand-induced rearrangement of Sirt2 active site-based inhibitors via molecular modeling and quantum mechanics calculations. Sci. Rep..

[31] Dubey A., Dotolo S., Ramteke P.W., Facchiano A., Marabotti A. (2018). Searching for Chymase Inhibitors among Chamomile Compounds Using a Computational-Based Approach. Biomolecules.

[32] Dubey A., Singh V., Doharey P.K., Sk M.P., Samanta S.K., Nema V., Sharma B., Varadwaj P.K., Sahoo A.K. (2021). Modulating catalytic activity of human topoisomerase II α enzyme by fluorescent gold nanoclusters. Int. J. Biol. Macromol..

[33] Li F.K.K., Rosell F.I., Gale R.T., Simorre J.P., Brown E.D., Strynadka N.C.J. (2020). Crystallographic analysis of Staphylococcus aureus LcpA, the primary wall teichoic acid ligase. J. Biol. Chem..

[34] Jones C.S., Sychantha D., Howell P.L., Clarke A.J. (2020). Structural basis for the O-acetyltransferase function of the extracytoplasmic domain of OatA from Staphylococcus aureus. J. Biol. Chem..

[35] Qiu X., Janson C.A., Smith W.W., Head M., Lonsdale J., Konstantinidis A.K. (2001). Refined structures of beta-ketoacyl-acyl carrier protein synthase III. J. Mol. Biol..

[36] Ju Y., Tong S., Gao Y., Zhao W., Liu Q., Gu Q., Xu J., Niu L., Teng M., Zhou H. (2016). Crystal structure of a membrane-bound l-amino acid deaminase from Proteus vulgaris. J. Struct. Biol..

[37] Bourne Y., Hasper A.A., Chahinian H., Juin M., De Graaff L.H., Marchot P. (2004). *Aspergillus niger* protein EstA defines a new class of fungal esterases within the alpha/beta hydrolase fold superfamily of proteins. Structure.

[38] Chang Z., Ansbacher T., Zhang L., Yang Y., Ko T.P., Zhang G., Liu W., Huang J.W., Dai L., Guo R.T. (2019). Crystal structure of LepI, a multifunctional SAM-dependent enzyme which catalyzes pericyclic reactions in leporin biosynthesis. Org. Biomol. Chem..

[39] Rose P.W., Prlic A., Bi C., Bluhm W.F., Christie C.H., Dutta S., Green R.K., Goodsell D.S., Westbrook J.D., Woo J. (2015). The RCSB Protein Data B ank: Views of structural biology for basic and applied research and education. Nucl. Acids Res..

[40] Sastry G.M., Adzhigirey M., Day T., Annabhimoju R., Sherman W. (2013). Protein and ligand preparation: Parameters, protocols, and influence on virtual screening enrichments. J. Comput. Aided Mol. Des..

[41] Schrödinger (2016). Release 2018-1: Schrödinger Suite 2018-1 Protein Preparation Wizard.

[42] Avogadro: An Open-Source Molecular Builder and Visualization Tool. Version 1.XX. http://avogadro.cc/.

[43] Marcus D.H., Donald E.C., David C.L., Tim V., Eva Z., Geoffrey R.H. (2012). Avogadro: An advanced semantic chemical editor, visualization, and analysis platform. J. Cheminf..

[44] Arima H., Ashida H., Danno G.-I. (2002). Rutin-enhanced antibacterial activities of flavonoids against Bacillus cereus and Salmonella enteritidis. Biosci. Biotechnol. Biochem..

[45] Miah M.A.T., Ahmed H.U., Sharma N.R., Ali A., Miah S.A. (1990). Antifungal activity of some plant extracts. Bangladesh J. Bot..

[46] Bishnu J., Sunil L., Anuja (2009). Antibacterial property of different medicinal plants: Ocimum sanctum, Cinnamomum zeylanicum, Xanthoxylum armatum and Origanum majorana. S. J. Sci. Eng. Technol..

[47] (1993). Methods for Dilution Antimicrobial Susceptibility Tests for Bacteria that Grow Aerobically Approved Standard, 3rd ed.

[48] (1992). Reference Method for Broth Dilution Antifungal Susceptibility Testing of Yeasts.

[49] Mosmann T. (1983). Rapid colorimetric assay for cellular growth and survival: Application to proliferation and cytotoxicity assays. J. Immunol. Methods.

